# Antiosteoarthritic Effect of Morroniside in Chondrocyte Inflammation and Destabilization of Medial Meniscus-Induced Mouse Model

**DOI:** 10.3390/ijms22062987

**Published:** 2021-03-15

**Authors:** Eunkuk Park, Chang Gun Lee, Seong Jae Han, Seung Hee Yun, Seokjin Hwang, Hyoju Jeon, Jeonghyun Kim, Chun Whan Choi, Siyoung Yang, Seon-Yong Jeong

**Affiliations:** 1 Department of Medical Genetics, Ajou University School of Medicine, Suwon 16499, Korea; jude0815@hotmail.com (E.P.); dangsunsang@naver.com (C.G.L.); yun41101@ajou.ac.kr (S.H.Y.); tjrwlshh@naver.com (S.H.); wjsgywn0315@ajou.ac.kr (H.J.); danbi37kjh@hanmail.net (J.K.); 2 Department of Biomedical Sciences, Ajou University Graduate School of Medicine, Suwon 16499, Korea; hsj2018@ajou.ac.kr; 3 Department of Pharmacology, Ajou University School of Medicine, Suwon 16499, Korea; 4 Natural Products Research Institute, Gyeonggi Institute of Science & Technology Promotion, Suwon 16229, Korea; cwchoi78@gmail.com

**Keywords:** osteoarthritis, alternative medicine, morroniside, anti-inflammatory effect

## Abstract

Osteoarthritis (OA) is a common degenerative disease that results in joint inflammation as well as pain and stiffness. A previous study has reported that *Cornus officinalis* (CO) extract inhibits oxidant activities and oxidative stress in RAW 264.7 cells. In the present study, we isolated bioactive compound(s) by fractionating the CO extract to elucidate its antiosteoarthritic effects. A single bioactive component, morroniside, was identified as a potential candidate. The CO extract and morroniside exhibited antiosteoarthritic effects by downregulating factors associated with cartilage degradation, including cyclooxygenase-2 (*Cox-2*), matrix metalloproteinase 3 (*Mmp-3*), and matrix metalloproteinase 13 (*Mmp-13*), in interleukin-1 beta (IL-1β)-induced chondrocytes. Furthermore, morroniside prevented prostaglandin E2 (PGE2) and collagenase secretion in IL-1β-induced chondrocytes. In the destabilization of the medial meniscus (DMM)-induced mouse osteoarthritic model, morroniside administration attenuated cartilage destruction by decreasing expression of inflammatory mediators, such as Cox-2, Mmp3, and Mmp13, in the articular cartilage. Transverse microcomputed tomography analysis revealed that morroniside reduced DMM-induced sclerosis in the subchondral bone plate. These findings suggest that morroniside may be a potential protective bioactive compound against OA pathogenesis.

## 1. Introduction

Osteoarthritis (OA) is a complex chronic disease and one of the most common types of arthritis, resulting in cartilage loss, pain, and stiffness [1,2]. The prevalence of OA is the most commonly observed among the elderly and obese, with a high risk of joint injuries [3]. Notably, the early onset of OA is initiated by mechanical stress in the articular cartilage, resulting in an excessive level of interleukin-1 beta (IL-1β) in the superficial zone of the cartilage [4,5]. Excessive levels of IL-1β trigger joint damage and inflammation via the upregulation of OA-inducing factors such as cyclooxygenase-2 (Cox-2) and matrix metalloproteinase (Mmp) [6,7]. These inflammatory mediators suppress collagen synthesis in chondrocytes following stimulation with prostaglandin E2 and collagenase secretion [8]. Recent studies have revealed that nuclear factor-κB (NF-κB) induces the expression of Cox-2, Mmp-3, and Mmp-13, while decreasing cartilage extracellular matrix (ECM) proteins, including collagen type II alpha 1 (Col2a1) synthesis [9,10]. Several pharmacological treatments against OA have focused on alleviating IL-1β mediated OA pathogenesis, including nonsteroidal anti-inflammatory drugs (NSAIDs), diacerein, and paracetamol [11,12,13]. However, these drugs have limitations with long-term administration owing to their serious side effects [14,15].

In recent years, natural plant-based treatments have been developed as alternatives for numerous diseases, with fewer side effects than conventional drugs [16,17]. Previous studies have shown that some plant extracts have preventive effects against OA pathogenesis [18,19]. *Cornus officinalis* (CO) of the dogwood genus has been found in Eastern Asia for more than 2000 years and has been employed as a traditional natural product for maintaining liver and kidney health [20]. Manu et al. have indicated the beneficial effects of CO on type 2 diabetes, neuroinflammation, and bone loss [21,22,23,24]. Recent studies have shown that the CO extract possesses potent anti-inflammatory action against oxidative stress in RAW 264.7 cells and decreases proinflammatory factors, such as IL-1β, interleukin-6 (IL-6), and tumor necrosis factor-alpha (TNF-α), in numerous types of inflammation [25,26,27]. Additionally, aqueous extracts of the CO fruit reportedly inhibit OA symptoms in a testosterone-deficient mouse model [28]. Phytochemical studies have demonstrated that herbal products contain a variety of bioactive components responsible for pharmacological activities against various diseases [29,30]. Numerous pharmaceutical bioactive components isolated from the CO extract have been identified, including morroniside, loganin, ursolic acid, and caffeic acid [31].

In the present study, we aimed to identify a single bioactive compound derived from the CO extract and evaluate its anti-OA protective effects in primary chondrocyte inflammation in vitro and destabilization of the medial meniscus (DMM) mouse model in vivo.

## 2. Results and Discussion

### 2.1. Cornus Officinalis (CO) Extract Reduces Mouse Primary Chondrocyte Inflammation

**Figure 1:**
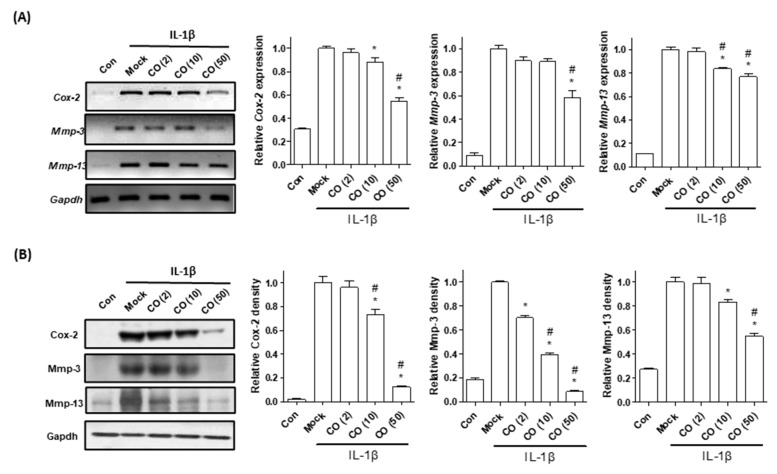
Effects of *Cornus officinalis* (CO) extract on chondrocyte inflammation. Mouse primary chondrocytes were incubated with interleukin 1 beta (IL-1β, 1 ng/mL) and cotreated with the CO extract (2, 10, and 50 μg/mL) for 48 h. (**A**) Mouse mRNA expressions of *Cox-2*, *Mmp-3*, and *Mmp-13* were examined by reverse-transcriptase polymerase chain reaction (RT-PCR, left) and quantitative RT-PCR (qRT-PCR, right). Glyceraldehyde 3-phosphate dehydrogenase (*Gapdh*) was used as an internal control. (**B**) Western blot analysis of Cox-2, Mmp-3, and Mmp-13 proteins in mouse primary chondrocytes. GAPDH was used as an internal control. The representative results are shown in the left panel, with quantitative data presented as a graph in the right panel. The results of triplicate experiments were examined by one-way analysis of variance (ANOVA, Tukey’s honestly significant difference post hoc test, analysis of variance). * *p* < 0.05 vs. Mock, # *p* < 0.05 vs. CO (2). Con; control, Mock; nontreated, CO; *Cornus officinalis* extract; IL-1β, interleukin-1 beta; Cox-2, cyclooxygenase-2 MMP-3, metalloproteinase 3; MMP-13, metalloproteinase 13; qRT-PCR; quantitative reverse transcriptase-polymerase chain reaction.

Primary chondrocyte inflammation induced by IL-1β treatment has been widely employed as an experimental model for investigating OA pathogenesis [32]. Chondrocytes are localized in the articular cartilage and play an important role in the modulation of ECM homeostasis [33]. During OA development, IL-1β initiates an inflammatory signaling cascade, inducing the expression of Cox-2, Mmp-3, and Mmp-13, resulting in OA pathogenesis [34,35]. In the present study, we determined whether the CO extract possessed anti-inflammatory effects against chondrocyte inflammation. Mouse primary chondrocytes were treated with IL-1β and coincubated with different concentrations of the CO extract (2, 10, and 50 μg/mL) for 48 h. The CO extract reduced IL-1β induced Cox-2, Mmp-3, and Mmp-13 mRNA and protein expression in chondrocytes when compared with the nontreated (Mock) group (Figure 1). Furthermore, the CO extract did not affect cell viability (Supplementary Figure S1A). These results indicate that the CO extract possesses anti-inflammatory activity against chondrocyte inflammation.

### 2.2. Identification of Morroniside Isolated from CO Extract

**Figure 2:**
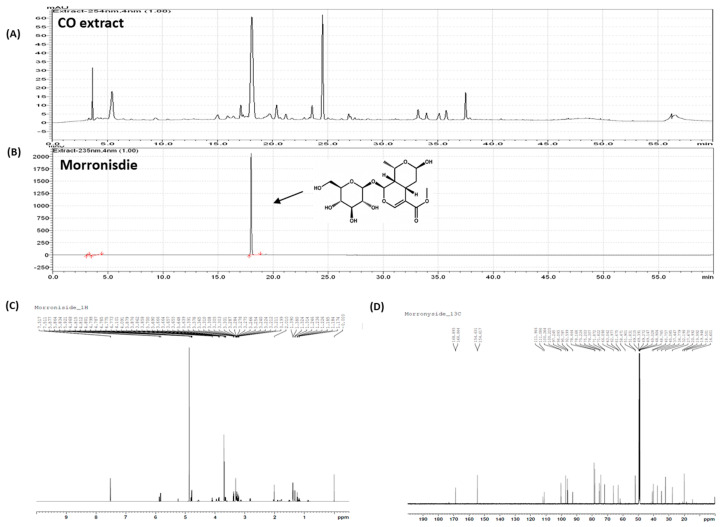
HPLC chromatogram of (**A**) total CO extract and (**B**) standard morroniside compound and (**C**) 1D and (**D**) 2D NMR spectrometry. HPLC, high-performance liquid chromatography; CO, *Cornus officinalis*; NMR, nuclear magnetic resonance.

The CO extract consists of several bioactive compounds with varying geological, cultivation, and climate differences [36,37]. Therefore, we performed a phytochemical screening of the CO extract fractions to elucidate the anti-inflammatory effects of the bioactive compounds on chondrocyte inflammation. High-performance liquid chromatography (HPLC) analysis of the CO extract is presented in Figure 2A. The anti-inflammatory effects of each fraction were determined by Cox-2 expression, a reliable marker for inflammatory signaling in chondrocytes [38]. Single bioactive compound(s) were fractionated by sequential fractionation (Supplementary Figure S2). Consequently, a specific bioactive compound morroniside was identified (Figure 2B) and confirmed by a combination of one- and two-dimensional nuclear magnetic resonance (NMR) spectrometry (Figure 2C,D).

### 2.3. Single Bioactive Compound, Morroniside, Attenuates Mouse Primary Chondrocyte Inflammation

**Figure 3:**
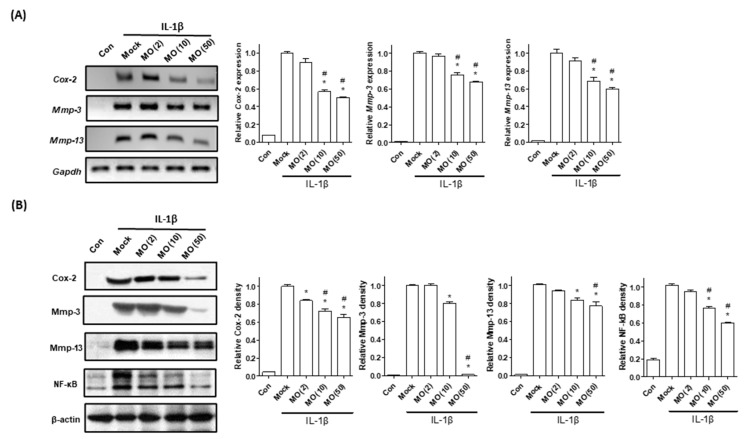
Effects of morroniside on chondrocyte inflammation. Mouse primary chondrocytes were incubated with IL-1β (1 ng/mL) and cotreated with morroniside (2, 10, and 50 μM) for 48 h. (**A**) mRNA expression levels of mouse *Cox-2*, *Mmp-3*, and *Mmp-13* were examined by RT-PCR (left) and qRT-PCR (right). (**B**) Protein levels of Cox-2, Mmp-3, Mmp-13, and NF-κB were analyzed by Western blotting. GAPDH was used as an internal control. The representative results are shown in the left panel, with quantitative data presented as a graph in the right panel. The results of triplicate experiments were examined by one-way ANOVA (Tukey’s honestly significant difference post hoc test, analysis of variance). * *p* < 0.05 vs. Mock, # *p* < 0.05 vs. MO (2). Con; control, Mock; nontreated, MO; morroniside; IL-1β, interleukin-1 beta; Cox-2, cyclooxygenase-2; MMP-3, metalloproteinase 3; MMP-13, metalloproteinase 13; qRT-PCR; quantitative reverse transcriptase-polymerase chain reaction.

Morroniside, an iridoid glycoside, is one of the main constituents of *C. officinalis* [39]. Phytochemical studies have shown that morroniside has various beneficial effects, including antidiabetic, antioxidative, cardioprotective, and antineuroinflammatory effects [40,41]. Based on previous studies, the anti-inflammatory effects of the CO extract could be exerted by morroniside. Therefore, we examined the anti-inflammatory effect of morroniside against chondrocyte inflammation. Primary cultured chondrocytes induced by IL-1β were treated with different concentrations of morroniside (2, 10, and 50 μM). In the present study, morroniside treatment attenuated inflammatory responses, such as Cox-2, Mmp-3, and Mmp-13, expression, when compared with those of the nontreated group (Figure 3). Additionally, morroniside treatment downregulated NF-κB protein levels. These results suggest that morroniside inhibits chondrocyte inflammation by reducing Cox-2, Mmp-3, Mmp-13, and NF-κB.

**Figure 4:**
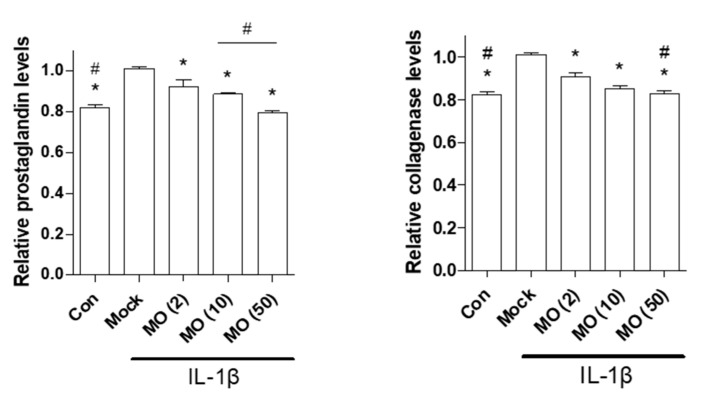
Effects of morroniside on prostaglandin E2 (PGE2) and collagenase secretion in IL-1β induced mouse primary chondrocytes. Cells were incubated with IL-1β (1 ng/mL) and cotreated with morroniside (2, 10, and 50 μM) for 48 h. PGE2 and collagenase levels in the total cell supernatant were determined. The results of triplicate experiments were evaluated by one-way ANOVA (Tukey’s honestly significant difference post hoc test). * *p* < 0.05 vs. Mock, # *p* < 0.05 vs. MO (2). Con; control, Mock; nontreated; MO, morroniside; IL-1β, interleukin-1 beta.

In OA inflammation, chondrocytes produce prostaglandin E2 (PGE2) and collagenase [42]. PGE2 plays a key role in the regulation of the inflammatory responses stimulated by Cox-2 expression [43]. In the synovial fluid, PGE2 and collagenase secretion induced by IL-1β participate in ECM degradation, resulting in mechanical stress in the subchondral region [44]. In the present study, morroniside treatment reduced IL-1β-mediated upregulation of PGE2 and collagenase levels in primary chondrocytes (Figure 4). These results indicate that morroniside prevents OA pathogenesis by reducing PGE2 and collagenase secretion.

### 2.4. Morroniside Administration Prevents DMM-Induced OA in Mice

**Figure 5:**
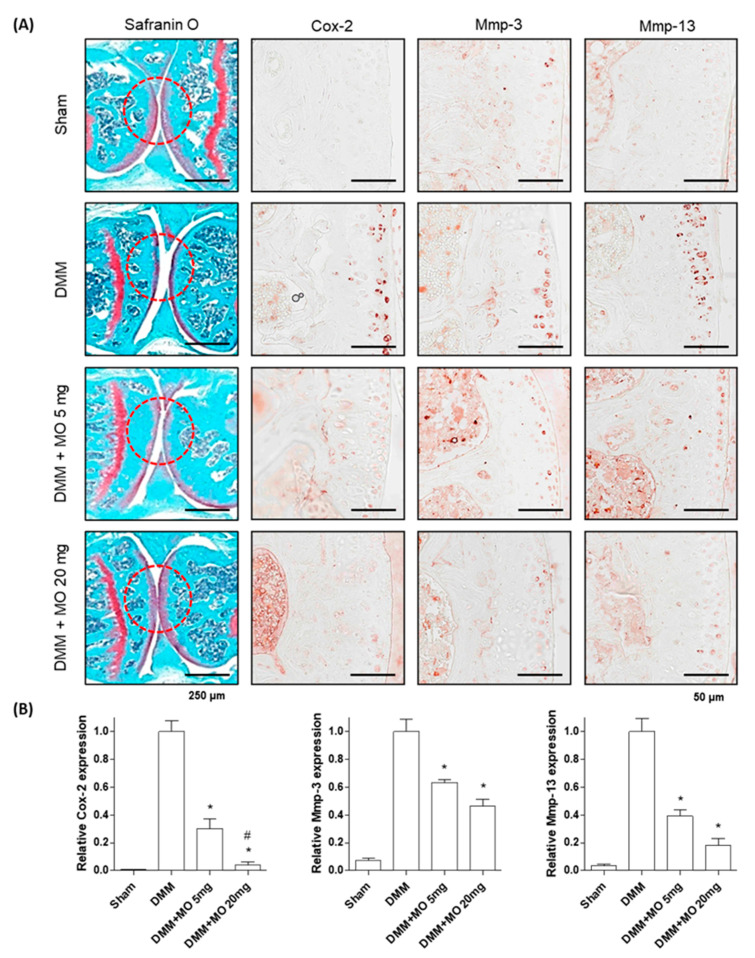
Effects of morroniside on DMM-induced OA pathogenesis in mice. Mice were treated with morroniside (5 and 20 mg/kg/day) for 10 weeks. (**A**) Articular cartilage sections were stained with safranin O and IHC for Cox-2, Mmp-3, and Mmp-13 was performed. Representative images were visualized by light microscopy. Red circle indicates the degeneration of the articular cartilage. (**B**) IHC images were quantified and determined using one-way ANOVA (Tukey’s honest significant difference post hoc test, analysis of variance). * *p* < 0.05 vs. DMM. # *p* < 0.05 vs. DMM+MO 5 mg. DMM, destabilization of the medial meniscus; OA, osteoarthritis; IHC, immunohistochemistry; Sham; sham-operated, MO; morroniside; Cox-2, cyclooxygenase-2; MMP-3, metalloproteinase 3; MMP-13, metalloproteinase 13. Scale bars: 50–250 μm.

**Figure 6:**
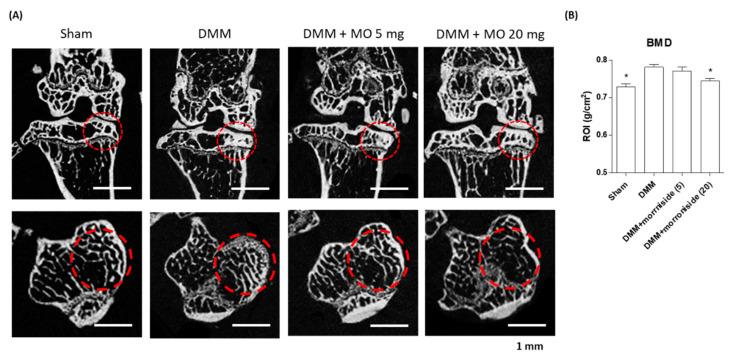
Effect of morroniside administration on DMM-induced osteophyte development. DMM-induced OA mice were treated with morroniside (5 and 20 mg/kg/day) for 10 weeks. (**A**) Representative micro-CT images of mouse knee joints reveal the density of the chondral bone plate. Red circle indicates osteophyte formation in response to inflammatory responses. (**B**) Bone mineral density (BMD) of proximal tibial epiphysis was analyzed using one-way ANOVA (Tukey’s honest significant difference post hoc test, analysis of variance). * *p* < 0.05 vs. DMM, DMM, destabilization of the medial meniscus; OA, osteoarthritis; CT, computed tomography; Sham; sham-operated, MO; morroniside.

The meniscus is a fibrocartilaginous tissue that extends mechanical integrity to the knee [45]. The loss of the meniscal function triggers increased mechanical stress in the articular cartilage, resulting in subchondral bone degeneration [46]. Furthermore, mechanical stress induces inflammatory responses in the articular cartilage via the secretion of IL-1β in the synovial region [47]. As the DMM mouse model mimics human OA, DMM mice have been used to investigate OA pathogenesis [48]. The DMM-induced OA model is characterized by the degeneration of the articular cartilage and osteophyte formation in response to inflammatory responses [49]. To investigate the antiosteoarthritic effect of morroniside in vivo, surgically destabilized medial meniscus mice were used to induce OA. Morroniside (5 and 20 mg/kg/day) was administered for 10 weeks. Morroniside treatment significantly reduced articular cartilage degeneration by decreasing inflammatory responses, including Cox-2, Mmp-3, and Mmp-13 expression, when compared with the nontreated DMM group (Figure 5). In addition, microcomputed tomography (micro-CT) images of the articular cartilage revealed that DMM mice presented osteophyte formation. However, morroniside inhibited DMM-induced sclerosis and cartilage degradation (Figure 6), indicating that morroniside prevents DMM-induced OA in vivo by reducing articular cartilage inflammation.

**Figure 7:**
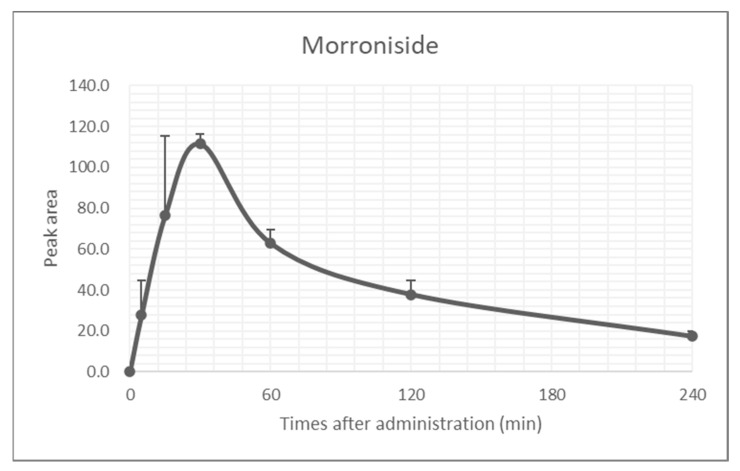
Metabolomic profile of morroniside in mice. Mean plasma concentration–time curves of morroniside in mice after a single administration (5 mg/kg). The data are presented mean ± standard error of the mean (SEM) (*n* = 3).

In terms of morroniside as a phytomedicine for antiosteoarthritic agents, metabolic profiles should be addressed to consider the bioavailability of the drug [50]. In the present study, we determined the bioavailability of morroniside in vivo. The blood concentrations of morroniside after a single administration were measured by HPLC analysis. Mice were orally administered morroniside (5 mg/kg) and blood was collected at 0, 5, 15, 30, 60, 120, and 240 min. According to a plasma clearance test, blood concentrations of morroniside peaked at 30 min, with almost complete removal observed within 240 min (Figure 7), indicating that morroniside was actively eliminated from the blood. These results suggest that morroniside might be a potent anti-inflammatory agent against OA pathogenesis.

## 3. Materials and Methods

### 3.1. Fractionation, Isolation, and Identification of Morroniside from CO Extract

Aerial parts of CO (1.4 kg) were dried in the shade, powdered, and added to 40 L of 70% ethanol (HPLC grade), twice at room temperature (each time for 2 days), and finally concentrated in a vacuum at 40 °C to yield 75.7 g of extract. The extracts were suspended in distilled water and Diaion HP-20 chromatography resin using gradient mixtures as eluents (100% H_2_O, 30% EtOH, 70% EtOH, EtOH fractions, F001–F004). Fraction F002 (30% EtOH) was separated by C18 MPLC using gradient mixtures as eluents (F011–F015). Morroniside was isolated from F012 by employing C18 MPLC. The structure was elucidated by a combination of 1D and 2D NMR spectrometry, as well as by comparison with reported literature (Figure 1) [51]. For in vitro and in vivo experiments, commercial morroniside was obtained from Sigma-Aldrich (St. Louis, MO, USA) and dissolved in distilled water.

Morroniside (1) white amorphous solid. ^1^H-NMR (CD_3_OD, 700 MHz) d: 1.31 (3H, d, *J* = 7.2 Hz, H-10b), 1.38 (3H, d, *J* = 7.2 Hz, H-10a), 1.75 (1H, ddd, *J* = 2.0, 4.0, 9.0 Hz, H-9b), 1.80 (1H, ddd, *J* = 2.0, 4.0, 9.0 Hz, H-9a), 1.88 (2H, m, H-6b), 2.00 (2H, m, H-6a), 2.82 (1H, dt, *J* = 4.0, 13.0 Hz, H-5a), 3.12 (1H, dt, *J* = 4.0, 13.0 Hz, H-5b), 3.35–3.41 (4H, m, H2, H-3, H-4, H-5), 3.70 (3H, s, OCH3), 3.87 (1H, dd, *J* = 2.0, 7.0 Hz, H-8a), 3.93 (1H, dd, *J* = 3.0, 7.0 Hz, H-8b), 4.24 (1H, dd, *J* = 2.0, 12.0 Hz, H6a), 4.44 (1H, dd, *J* = 2.0, 12.0 Hz, H-6b), 4.78 (1H, d, *J* = 4.0 Hz, H-7), 4.80 (1H, d, *J* = 8.0 Hz, H-1), 5.82 (1H, d, *J* = 8.6 Hz, H-1a), 5.86 (1H, d, *J* = 9.0 Hz, H-1b), 7.51 (1H, s, H-3);

^13^C-NMR (CD_3_OD, 175 MHz) d: 19.8 (C-10), 27.3 (C-5b), 31.9 (C-5a), 34.5 (C-6b), 37.1 (C-6a), 39.7 (C-9a), 40.4 (C-9b), 51.8 (OCH_3_), 62.6 (C-6), 65.8 (C-8b), 71.4 (C-2), 74.0 (C4), 74.9 (C-8a), 77.8 (C-5), 78.3 (C-3), 95.5 (C-7), 96.9 (C-1), 99.9 (C1), 110.7 (C-4), 154.4 (C-3), 168.7 (CO).

### 3.2. Mouse Chondrocyte Primary Culture and the Destabilization of Medial Meniscus (DMM) Mouse Model

All animal experiments in the present study were approved by the Institutional Animal Care and Use Committee of Ajou University School of Medicine (2016–0062), and experiments were conducted in accordance with the guidelines of the committee. Primary chondrocytes were isolated from the knee joint of mice as previously described [52]. Briefly, the articular cartilage of the knee joint from 5-day-old mice was dissected and incubated with a digestive solution (Dulbecco’s modified Eagle’s medium (Invitrogen, Carlsbad, CA, USA) containing 1% collagenase type II (Sigma), 0.5% trypsin EDTA, and 1% penicillin/streptomycin) for 2 h. Digestive suspensions were filtered through a Corning^®^ 40 μm cell strainer (Sigma), followed by centrifugation at 1500 rpm. Cell pellets were resuspended in DMEM, supplemented with 10% fetal bovine serum (Invitrogen) and 1% penicillin/streptomycin. Next, the cells (3 × 10^6^ cells/well) were seeded in 6-well plates and maintained at 37 °C in a humidified incubator with 5% CO_2_. For the DMM-induced OA mouse model, 12-week-old male C57BL/6 mice were anesthetized with tiletamine/zolazepam (Zoletil; Vibrac Laboratories, Carros, France), and the medial meniscus in the left articular cartilage was surgically removed. After surgery, mice were administered morroniside (5 mg/kg and 20 mg/kg) by oral gavage for 10 weeks. At the end of the experiment, the mice were anesthetized with Zoletil and evaluated bone mineral density (BMD) of knee joint using PIXI-mus bone densitometer with the on-board PIXI-mus software (GE Lunar, Madison, WI, USA). At the end of the experiment, the left articular cartilage was subjected to histological analysis.

### 3.3. Cell Viability and Cell Supernatant Analysis

Primary chondrocytes (1 × 10^5^ cells/well) were seeded overnight in 96-well plates at 37 °C and treated with different concentrations of the CO extract (2, 10, and 50 μg/mL) or morroniside (2, 10, and 50 μM) for 48 h. Cell viability was determined using the D-Plus™ CCK cell viability assay kit (Donginbiotech, Seoul, Korea) in accordance with the manufacturer’s instructions. To analyze PGE2 and collagenase levels secreted by the chondrocytes, total cell supernatants were concentrated with Vivaspin^®^ 2 Centrifugal Concentrator (Sartorius, Göttingen, Germany) and assessed using the Prostaglandin E2 Parameter Assay Kit (R&D Systems, Minneapolis, MN, USA) and EnzChek™ Gelatinase/Collagenase Assay Kit (Invitrogen), respectively, in accordance with the manufacturer’s instructions.

### 3.4. Reverse Transcriptase-Polymerase Chain Reaction (RT-PCR) and Quantitative RT-PCR (qRT-PCR)

Total RNA from mouse primary chondrocytes was isolated using TRIzol reagent (Invitrogen) according to the manufacturer’s instructions. Complementary DNA (cDNA) was synthesized using RevertAid™ H Minus First Strand cDNA synthesis kit (Fermentas, Hanover, NH, USA), RT-PCR was performed using the HiPi Plus 5× PCR MasterMix (ELPIS Biotech, Daejeon, Korea), and the qRT-PCR was performed using SYBR Green I qPCR kit (TaKaRa, Shiga, Japan), according to the manufacturer’s instructions. The gene-specific primers for inflammatory responses in primary chondrocytes were as follows: 5′-GGT CTG GTG CCT GGT CTG ATG AT-3′ and reverse 5′-GTC CTT TCA AGG AGA ATG GTG C-3′ for mouse *Cox-2*; forward 5′-CTG TGT GTG GTT GTG TGC TCA TCC TAC-3′ and reverse 5′-GGC AAA TCC GGT GTA TAA TTC ACA ATC-3′ for mouse *Mmp-3*; forward 5′-TGA TGG ACC TTC TGG TCT GGC-3′ and reverse 5′-CAT CCA CAT GGT TGG GAA GTT CTG-3′ for mouse *Mmp-13*; forward 5′-TCA CTG CCA CCC AGA C-3′ and reverse 5′-TGT AGG CCA TGA GGT CCA C-3′ for mouse *GAPDH*. The qRT-PCR protocol was as follows: 95 °C for 10 min, followed by 40 cycles of 95 °C for 5 s, 60 °C for 30 s, and 72 °C for 30 s, and finally a terminal melting step of 72 °C to 95 °C for 2 min (5 s per degree). Gene expression was normalized to *GAPDH* expression. Results were determined using the 2^−ΔΔCt^ method, and the fold change was expressed by comparison with the untreated control group.

### 3.5. Western Blot Analysis and Antibodies

Cells were washed thrice with phosphate-buffered saline and then lysed with radioimmunoprecipitation assay (RIPA) buffer (BIOSESANG, Seongnam, Gyeonggi-do, Korea) containing phenylmethylsulfonyl fluoride (PMSF) cocktail (Sigma) and phosphatase inhibitor cocktail (Sigma). Cell lysates were separated by sodium dodecyl sulfate-polyacrylamide gel electrophoresis (SDS-PAGE) and transferred to polyvinylidene fluoride membranes (Merck Millipore, Burlington, MA, USA). To evaluate alterations in the levels of proteins associated with inflammatory responses, cell lysates and mouse femoral bone slides were analyzed using appropriate primary antibodies (mouse Cox-2 (sc-1745, Santa Cruz, Dallas, TX, USA), mouse Mmp-3 (ab52915, Abcam, Cambridge, UK), mouse Mmp-13 [ab51072, Abcam], NF-κB p65 (#8242, Cell Signaling Technology, Danvers, MA, USA), and β-actin (sc-47778, Santa Cruz)). The bands of three independent Western blots and IHC images were quantified by ImageJ (Version 1.53g) software (NIH, Rockville, MA, USA).

### 3.6. Histology and Micro-CT Analysis

Mouse knee joints were fixed with 4% paraformaldehyde for 48 h and then decalcified with 0.5 M ethylenediaminetetraacetic acid (EDTA, pH 8.0) for 2 weeks. Bone tissues were embedded in paraffin and sectioned (4 μm) using a rotary microtome. Tissue sections were stained with safranin O for assessment of the subchondral region and visualized by optical microscopy (Leica, Wetzlar, Germany). For micro-CT analysis, a paraformaldehyde-fixed femoral bone was scanned using the high-energy spiral scan micro-CT (Skyscan 1173; Bruker, Billerica, MA, USA). Two-dimensional CT images were reconstructed using the CTvox Software version 3.2 (Bruker).

### 3.7. Statistical Analysis

All results are presented as the mean ± standard error of the mean (SEM), and statistical analysis was performed using GraphPad Prism 5.0 (GraphPad, San Diego, CA, USA). Multiple comparisons between groups were evaluated by one-way analysis of variance (ANOVA), followed by Tukey’s honestly significant difference (HSD) post hoc test. A probability value less than 0.05 (*p* < 0.05) was considered statistically significant.

## 4. Conclusions

In the present study, we examined the antiosteoarthritic effect of a single bioactive component morroniside isolated from the CO extract. This bioactive compound was found to be responsible for the anti-inflammatory effects mediated by the CO extract. Morroniside reduced IL-1β-induced primary cultured chondrocyte inflammation by decreasing the expression of Cox-2, Mmp3, and Mmp13, as well as PGE2 and collagenase secretion. In the DMM mouse OA model, morroniside administration ameliorated cartilage degeneration, inflammatory responses, and osteophyte formation. These findings suggest that morroniside may be a putative pharmacological agent against OA.

## Data Availability

The data presented in this study are available on request from the corresponding author.

## Supplementary Materials

The following are available online at https://www.mdpi.com/1422-0067/22/6/2987/s1, Figure S1: Effects of *Cornus officinalis* (CO) extract and morroniside on mouse primary chondrocytes; Figure S2: Fractionation and isolation of the bioactive component from CO extract.
