# Supplementary material for: Antiosteoarthritic Effect of Morroniside in Chondrocyte Inflammation and Destabilization of Medial Meniscus-Induced Mouse Model
Source: Int J Mol Sci. 2021 Mar 15;22(6):2987. doi: 10.3390/ijms22062987 (PMC7999654; doi:10.3390/ijms22062987)
Supplement: Supplementary file 1 [file ijms-22-02987-s001.pdf]

Antiosteoarthritic Effect of Morroniside in Chondrocyte Inflammation and Destabilization of Medial Meniscus-Induced Mouse Model

Eunkuk Park, Chang Gun Lee, Seong Jae Han, Seung Hee Yun, Seokjin Hwang, Hyoju Jeon, Jeonghyun Kim, Chun Whan Choi, Siyoung Yang and Seon-Yong Jeong

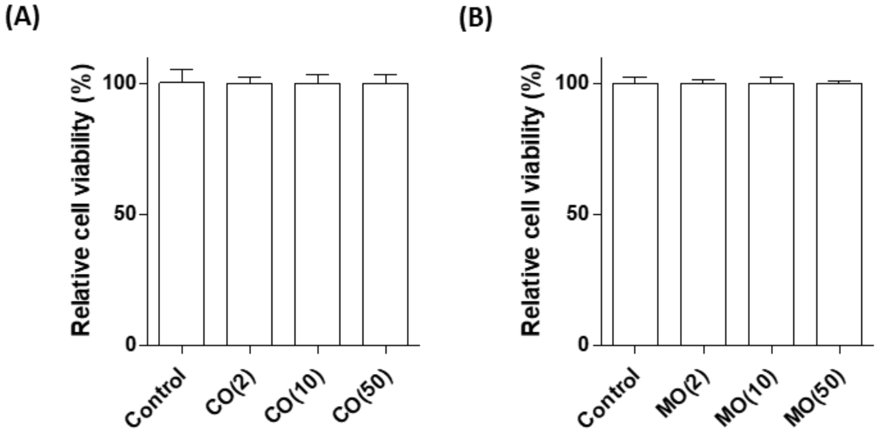

Supplementary Figure S1. Effects of *Cornus officinalis* (CO) extract and morroniside (MO) on mouse primary chondrocytes. Primary chondrocytes were incubated with different concentrations of (A) CO extract (0, 2, 10, and 50 μg/mL) and (B) MO (0, 2, 10, and 50 μM) for 48 h. Cell viability was examined by D-Plus™ CCK cell viability assay kit.

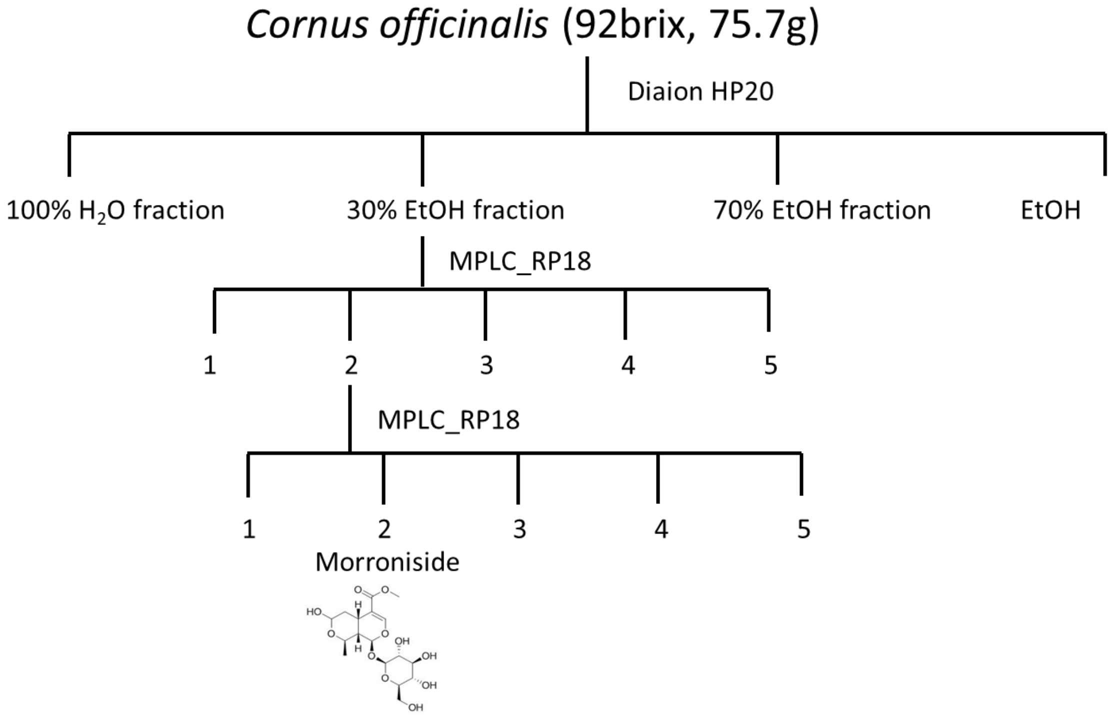

Supplementary Figure S2. Fractionation and isolation of the bioactive component from *Cornus officinalis* extract.
